# Bilateral Sternalis Muscles: The Clinical Significance of This Rare Discovery

**DOI:** 10.7759/cureus.60507

**Published:** 2024-05-17

**Authors:** Annie Shi Ru Li, Michelle Sue, Peter Lombardi, Harun S Bola, Danielle C Bentley

**Affiliations:** 1 Faculty of Arts & Science, University of Toronto, Toronto, CAN; 2 Faculty of Medicine, Department of Surgery, Division of Anatomy, University of Toronto, Toronto, CAN

**Keywords:** medical education, human dissection, anterior thoracic wall, cadaveric studies, accessory muscles, vestigial muscles, sternalis muscle variations, bilateral sternalis muscle, sternalis muscle

## Abstract

This case report explores the physical characteristics and clinical significance of the sternalis muscle, an uncommon anatomical variation of the anterior thoracic wall. If present, the sternalis muscle may distort diagnostic images and can be associated with incorrect interpretation of such medical images, misdiagnoses, and even surgical complications. As such, enhancing clinicians’ knowledge of this muscle and improving its recognition is of the utmost importance. In this case report, a rare bilateral sternalis muscle that was discovered during an educational human cadaveric dissection of a 73-year-old Black male is described. The right sternalis muscle fibres extended from the mid-sternal level to the right sternocostal arch, measuring 11.5 cm in length and 3.4 cm at its largest width. In contrast, the smaller left sternalis muscle fibres measured only 5.6 cm in length and 1.2 cm at its greatest width. This rare bilateral presentation of the sternalis muscle is documented in approximately one-third of all reported sternalis cases with an associated estimated prevalence as low as 1.7% among the general population. Serving as a reminder of the intricate anatomical complexities that continue to challenge and intrigue medical professionals, this report advocates for continued education of anatomical variations to enhance patient care and medical practices.

## Introduction

In the early 17th century, professor of anatomy Barthelemy Cabrol (also known as Cabrolius) became the first of many to report a previously unknown muscle superficial to the pectoralis major muscle on the anterior aspect of the thorax [1]. Over the next four centuries, this intriguing muscle has been called many names, such as musculus sternalis, rectus sternalis, and parasternalis, all of which are derived from the muscle’s proximity to the sternum [1,2].

The sternalis muscle is now a well-documented, albeit uncommon, anatomical variation of the anterior thoracic musculature, present in approximately 7.8% of the general population [1]. The sternalis muscle is most often discovered during either cadaveric dissection or diagnostic imaging and typically presents as a unilateral band of muscular fibres on the right side of the body [1], with rare instances of either left-side muscle fibres or bilateral muscle fibres.

Despite recent investigations, the exact embryological origin and developmental sequence of the sternalis muscle remain to be elucidated and are highly debated. Current theories using cadaveric and fetal studies propose that the sternalis muscle may arise as a derivative of an adjacent muscle, with the pectoralis major, the sternocleidomastoid, and the rectus abdominis muscles commonly suggested as candidates [1-3]. In contrast, comparative animal studies have led others to hypothesize that the sternalis muscle may be similar to the panniculus carnosus, a ventral thoracic wall muscle common to lower vertebrates [1,3]. More research is required before the likelihood of either hypothesis can be established.

Regardless of embryological origin, the sternalis muscle is hypothesized to be either an accessory or vestigial muscle without a known functional significance to the human body [1,3]. However, it does hold clinical significance in healthcare settings. As it is unknown to many patient-facing clinicians and technologist team members, the sternalis muscle is often mistaken for a benign or malignant growth on the anterior chest wall [2,4]. The presence of this muscle can subsequently influence the reading and/or interpretation of mammograms [5] and electrocardiograms (ECGs) [2,4,6], as well as surprise unexpecting surgeons during breast or thoracic surgeries [3]. Such confusion may lead to clinical misdiagnoses, resulting in unnecessary patient confusion and stress. As such, it is of the utmost importance that healthcare professionals in clinical settings enhance their awareness of this unique muscle to ensure accurate diagnoses, prevent complications, and minimize patient distress. Emphasizing the significance of this matter is critical, as it directly impacts patient well-being and medical precision.

## Case presentation

This presentation of an Anatomy Clinical Case Report has been declared exempt from the University of Toronto Research Ethics Board (standing letter of exemption for donor case reports of less than three donors for which no identifiable information is published).

During the practical dissection component within an advanced undergraduate anatomy course, bilateral sternalis muscle bellies were discovered on a 73-year-old Black male formalin-fixed donor. The medically documented causes of death were congestive heart failure and atrial fibrillation. The donor had well-defined and maintained skeletal musculature along with other intriguing anatomical features, including a right-sided direct inguinal hernia, a basilar artery fenestration, an enlarged heart with a coronary artery bypass, and an accessory left testicular vein. Students were guided through in-laboratory tasks via Grant’s Dissector, 17th edition [7].

**Figure 1:**
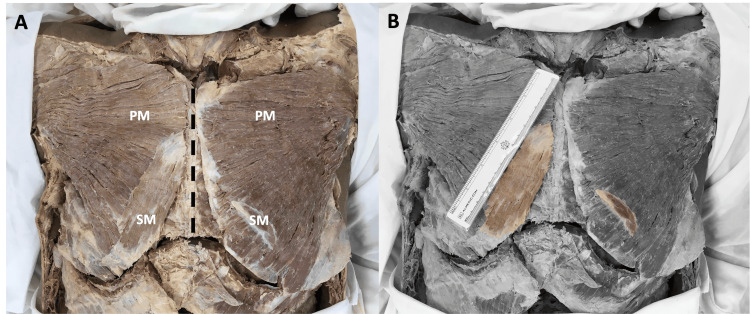
Anterior view of the thorax. A) The bilateral sternalis muscle (SM) fibres can be seen running perpendicular to the lower fibres of the bilateral pectoralis major (PM) muscles. B) Grayscale image with the bilateral sternalis muscles colourized for enhanced clarity of muscular borders.

During the removal of skin in preparation for the task of opening the anterior thoracic wall, two unexpected muscle bellies were discovered. First, a right sternalis muscle belly was discovered superficial to the corresponding pectoralis major muscle (Figure 1A). These muscle fibres ran perpendicular to the pectoralis major and parallel to the long axis of the sternum. The right sternalis muscle fibres extended from the mid-sternal level to the right sternocostal arch, with the muscle belly presenting as a flat, ribbon-like band measuring 11.5 cm in length and 3.4 cm at its largest width (Figure 1B). Following the unexpected discovery of the right sternalis muscle belly, a subsequent exploration began for a possible left sternalis muscle. However, the identification of the left sternalis muscle posed greater difficulty due to its significantly smaller muscle belly size, measuring only 5.6 cm in length and 1.2 cm at its greatest width (Figure 1B). Together, these were identified as bilateral sternalis muscles, a rare presentation of the already-rare sternalis muscle, with an estimated population prevalence as low as 1.7% [2].

## Discussion

Through cadaveric studies, the overall prevalence of the sternalis muscle, regardless of laterality, amongst the general population is estimated to be approximately 7.8% [1]. This overall estimate can be stratified specific to the presented case by both race and genetic sex, with a slightly greater prevalence among the Black population (8.4%) [2] and a slightly lesser prevalence among genetic males (7.5%) [1]. Interestingly though, the bilateral presentation of the sternalis muscle, as described in this clinical case report, is a rare finding. While some researchers have estimated that as many as one-third of reported sternalis cases have bilateral muscle fibres [1,8], others have estimated a prevalence rate as low as 1.7% [2]. Regardless, the presented case report has described a rare anatomical variant of an underappreciated muscle, often unknown to healthcare professionals [9,10].

The unfamiliarity of the sternalis muscle to healthcare professionals was recently examined. Specifically, when physicians (175 intern doctors, 26 surgery residents, and 28 radiology residents) were asked to identify the sternalis muscle from a CT image and an anatomical figure of the chest anatomy, only 35.7% of the radiology residents and 3.8% of the surgery residents could correctly identify the sternalis muscle from the two images [9]. In contrast, all the intern doctors either left their answers blank or answered incorrectly [9]. This study was the second of two published reports to directly examine the awareness of the sternalis muscle among healthcare professionals. The first study to examine physicians’ awareness was conducted in 1999 among 65 physicians and medical students from diverse disciplines, including general surgery, plastic surgery, and radiology; it also revealed a near-universal unfamiliarity with the sternalis muscle among the respondents [10].

Anatomical variants, such as the sternalis muscle and the laterality of its presentation, can be used as pedagogic tools. They can be used to initiate student-led discussions on the associated developmental origin and spark debate on possible function(s), while simultaneously encouraging a more comprehensive appreciation of anatomical variations [11]. Overall, augmented focus on the sternalis muscle in scientific publications and educational resources can help medical professionals become more informed and better equipped to accurately identify and interpret cases involving this variation of the anterior thoracic wall.

Although the sternalis muscle is not associated with any noteworthy patient symptoms, it does hold clinical significance. The sternalis muscle’s presence may lead to potential misdiagnoses or false-positive findings. For example, the presence of the sternalis muscle may mimic pathologies such as breast masses or lymph node enlargement, potentially leading to cancer-related investigations [11]. Moreover, the sternalis muscle may interfere with ECG interpretations, potentially leading to improper management of cardiac conditions [2,11].

Specific to surgery, understanding the presence and variations of the sternalis muscle can guide surgical planning, particularly in breast or chest wall surgeries [3,12], where accurate knowledge of anatomical structures is crucial for successful outcomes. Pioneering plastic surgeons have suggested an innovative functionality of the sternalis muscle as a muscle flap in reconstructive and/or plastic surgeries of the head, the neck, and the anterior thoracic wall [12,13]. As such, surgeons should acquaint themselves with the sternalis muscle and its variety of presentations, not only to avoid unintended injury or complications during anterior chest wall procedures but also to remain at the forefront of potential surgical advancements.

## Conclusions

This case report describes a rare bilateral presentation of the sternalis muscle discovered during an educational dissection of a 73-year-old Black male human cadaver, highlighting some of its clinical significance and potential impact on medical practices. The muscle’s infrequent prevalence, deceptive resemblance to other pathologies, and potential influence on both diagnostic tests and surgical procedures necessitate improved awareness among both patient-facing clinicians and technologist team members.
